# *Escovopsioides* as a fungal antagonist of the fungus cultivated by leafcutter ants

**DOI:** 10.1186/s12866-018-1265-x

**Published:** 2018-10-10

**Authors:** Julio Flavio Osti, Andre Rodrigues

**Affiliations:** 1 Department of Biochemistry and Microbiology, São Paulo State University (UNESP), Rio Claro, Brazil; 2 Center for the Studies of Social Insects, São Paulo State University (UNESP), Rio Claro, Brazil

**Keywords:** Hypocreales, Attine ants, *Escovopsis*, Symbiosis

## Abstract

**Background:**

Fungus gardens of fungus-growing (attine) ants harbor complex microbiomes in addition to the mutualistic fungus they cultivate for food. Fungi in the genus *Escovopsioides* were recently described as members of this microbiome but their role in the ant-fungus symbiosis is poorly known. In this study, we assessed the phylogenetic diversity of 21 *Escovopsioides* isolates obtained from fungus gardens of leafcutter ants (genera *Atta* and *Acromyrmex*) and non-leafcutter ants (genera *Trachymyrmex* and *Apterostigma*) sampled from several regions in Brazil.

**Results:**

Regardless of the sample locality or ant genera, phylogenetic analysis showed low genetic diversity among the 20 *Escovopsisoides* isolates examined, which prompted the identification as *Escovopsioides nivea* (the only described species in the genus). In contrast, one *Escovopsioides* isolate obtained from a fungus garden of *Apterostigma megacephala* was considered a new phylogenetic species. Dual-culture plate assays showed that *Escovopsioides* isolates inhibited the mycelium growth of *Leucoagaricus gongylophorus*, the mutualistic fungus cultivated by somes species of leafcutter ants. In addition, *Escovopsioides* growth experiments in fungus gardens with and without ant workers showed this fungus is detrimental to the ant-fungus symbiosis.

**Conclusions:**

Here, we provide clues for the antagonism of *Escovopsioides* towards the mutualistic fungus of leafcutter ants.

**Electronic supplementary material:**

The online version of this article (10.1186/s12866-018-1265-x) contains supplementary material, which is available to authorized users.

## Background

Fungus-growing ants in the tribe Attini are found only on the American continent [1]. These insects cultivate fungi as the main food source for the colony [2]. As a subgroup within the higher attines, leafcutter ants in the genera *Atta* and *Acromyrmex* use fresh leaves and flowers as substrate to nourish the fungus cultivar. Leafcutter ants cultivate two phylogenetic clades of fungi including *Leucoagaricus gongylophorus* (Basidiomycota: Agaricales) [3]. In turn, the fungal partner is cultivated on fragments of plant substrate processed by the ants in a structure named “fungus garden” [2]. Due to the high amount of leaves that leafcutter ants collect, these insects are considered agricultural pests, causing serious damage to several crops [4].

Contrary to what was proposed by Weber [2], *L. gongylophorus* is not the only active microorganism in the fungus garden. In fact, a diverse and rich microbial community consisting of bacteria, yeasts, and filamentous fungi are also found in this substrate [5–7]. Some of these microorganisms can compete for nutrients with the mutualistic fungus [8], others act as parasites of the fungal cultivar [9]. In addition, there are beneficial microbes in the fungus gardens, which may help the mutualistic fungus against pathogens [10]. Actinobacteria present on the workers’ cuticles also assist in the protection of the colony against invading microorganisms [11–13].

Currie and colleagues [14] showed that fungi in the genus *Escovopsis* (Ascomycota: Hypocreales) cause negative impacts on attine ant colonies. Multiple lines of evidence suggest that *Escovopsis* is a specialized parasite of the ant cultivar, using both chemical and physical mechanisms to attack its host [9, 15, 16]. *Escovopsis* shares an ancient evolutionary history with the mutualistic fungus and the ants, indicating that the origin of this parasite occurred concomitantly with the beginning of the ant-fungus association, about 50–65 million years ago [17].

Recently, fungi in the genus *Escovopsioides* (Ascomycota: Hypocreales) were described from fungus gardens of the leafcutter ants *Acromyrmex niger*, *Acromyrmex subterraneus molestans* and *Acromyrmex subterraneus subterraneus* [18]. Also, this fungus was found in 24% to 72% of the sampled colonies in different localities in Brazil [18–20]. *Escovopsioides* shares some morphological characteristics with *Escovopsis* but differ in others such as terminal vesicles with long (lageniform) phialides and hyphae with aleuroconidia. Molecular analysis indicates that *Escovopsioides* is phylogenetically related to *Escovopsis* [18]. The discovery of this fungus launched new research windows on the attine ant-fungus symbiosis. First, nothing is known about the diversity and taxonomy of *Escovopsioides*. So far, only one species (*E. nivea*) has been described from a very narrow geographical range (“Mata do Paraíso”, Viçosa, state of Minas Gerais, Brazil); however, it is likely that other species in the genus still await discovery. Second, if *Escovopsioides* is an antagonist of the mutualistic fungus as suggested by Varanda-Haifig et al. [16], new opportunities will rise to study the dynamics of the evolution of additional detrimental fungi to the fungus gardens of attine ants. Third, it is not clear whether *Escovopsioides* can cause detrimental effects to fungus gardens of attine ants, or whether the ant workers protect the fungus gardens against *Escovopsioides*.

We obtained several *Escovopsioides* isolates from fungus gardens of different associated attine ant genera (*Atta*, *Acromyrmex*, *Trachymyrmex* and *Apterostigma*) and posed the following questions: (i) given that *Escovopsioides* was described from fungus gardens of *Acromyrmex* spp., do isolates of this fungus found on colonies of other genera of attine ants form a monophyletic clade?, (ii) are *Escovopsioides* from different associated attine ant species antagonists of the *L. gongylophorus* and the fungus gardens?, and (iii) is *Escovopsioides* capable to overcome the protective roles of the ant workers? We show that *Escovopsioides* associated with a wide range of ant species from various geographical areas belong to the same species (*E. nivea*). Our results also support previous findings [16] of the antagonism of this fungus towards the mutualistic cultivar of leafcutter ants.

## Results

### *Escovopsioides* phylogeny

**Fig. 1:**
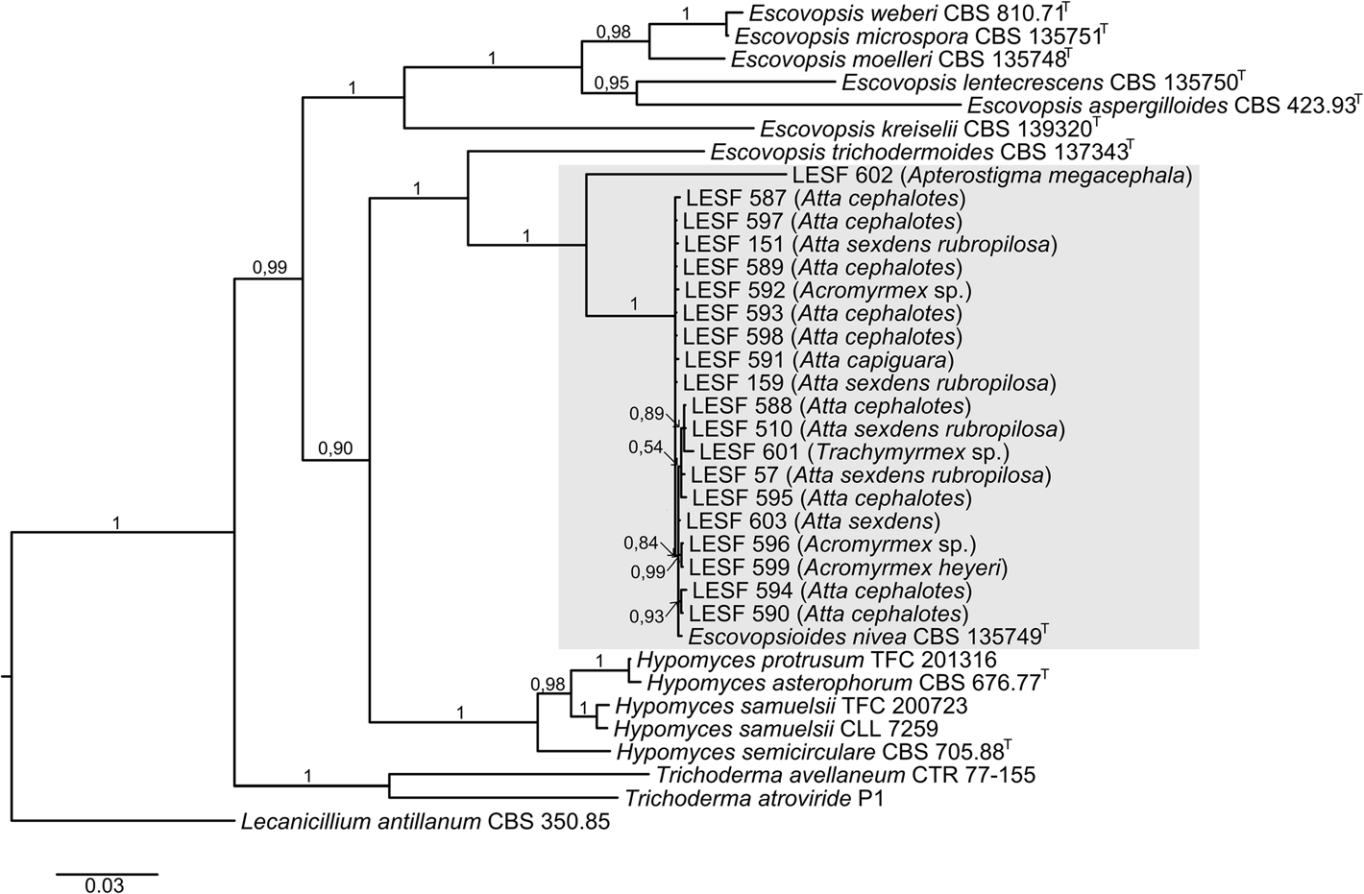
Phylogeny of *Escovopsioides* isolates obtained from fungus-growing ants. The tree was reconstructed under Bayesian Inference (BI) using concatenated sequences of *tef1* and LSU genes as well as ITS. Sequences of type strains of *Escovopsis* spp. (represented by ^T^) and additional hypocrealean fungi (outgroup) were retrieved from GenBank (Additional file 1: Table S1). Values on branches are posterior probabilities. The entire alignment is composed of 2112 bp in length. The substituion models GTR + G for *tef1* and GTR + I + G for ITS and LSU were used in the BI analysis. *Escovopsioides* species are indicated by LESF Ids and associated ant species are indicated in parentheses. All other sequences are followed by the culture collection accessions

Our analysis resolved a single, well-supported monophyletic clade of *Escovopsiodes*, with one isolate falling just outside of this clade, namely isolate *Escovopsioides* sp. LESF 602 (Fig. 1). We observed low genetic diversity in the main clade, comprising isolates associated with the higher-attine ant genera *Atta*, *Acromyrmex* and *Trachymyrmex* along with the type species, *E. nivea* CBS 135749^T^. A low intraspecific variation (99.1 to 100% similarity) among *Escovopsioides* isolates of higher-attines was observed in the *tef1* marker. Interesting to note, the genetic similarity in the *tef1* gene was observed for isolates from distant geographic regions, since *Escovopsioides* isolates obtained from colonies separated by large distances were similar, such as Rio Grande do Sul and Bahia states (Fig. 1). The low intraspecific variation was also observed in the ITS region (99.4 to 100% of similarity). The LSU sequences were identical among 20 out of 21 sequences. On the other hand, *Escovopsioides* sp. LESF 602 obtained from fungus gardens of *Apterostigma megacephala* presented similarity between 90 to 91% in relation to the other isolates considering the marker *tef1*; 91% to 92% for ITS and 98% of similarity for LSU. This isolate stands out in the phylogeny as a separate branch and indicates a not yet described phylogenetic species (Fig. 1).

### Dual-culture bioassays

A total of 12 out of 14 evaluated fungi (13 *Escovopsioides* and 1 *Escovopsis*) significantly inhibited the mycelial growth of *L. gongylophorus* FF2006 after 10 days (two-way mixed ANOVA, *P* < 0.009, Table 1). Except for *E. nivea* LESF 596 and *Escovopsioides* sp. LESF 602, the remaining *Escovopsioides* isolates inhibited the growth of this fungus between the 3rd and 7th days of experiment (Bonferroni test, *P* < 0.01). Inhibition of the mutualistic fungus by isolates LESF 596 and LESF 602 was only observed between the 7th and 10th day (Bonferroni test, *P* < 0.01, Table 1). On the other hand, significant inhibition of mycelial growth of *L. gongylophorus* by *Escovopsis* was observed on the 2nd day of the experiment (Bonferroni test, *P* < 0.01, Table 1). All *Escovopsioides* isolates began to significantly inhibit *L. gongylophorus* when contact between colonies of the two fungi occurred, when the mutualistic fungus generally acquired a brown coloration.

**Table 1:** Mycelial growth (mean areas ± standard errors, in cm^2^) and interval of inhibition of *Leucoagaricus gongylophorus*, the fungus cultivated by the leafcutter ant *Atta sexdens rubropilosa*. T: type species of *Escovopsioides nivea*

Isolate ID	Fungi	CG_d10_	FG_d10_	Int-In	F value	*P* value
LESF 19	*Escovopsis* sp.	8.97 ± 0.07	3.43 ± 0.07	0–2	320.43	< 0.001
LESF 601	*Escovopsioides nivea*	8.97 ± 0.07	4.36 ± 0.12	3–5	127.26	< 0.001
LESF 590	*Escovopsioides nivea*	9.10 ± 0.13	5.20 ± 0.10	3–5	103.81	< 0.001
CBS 135749^T^	*Escovopsioides nivea*	8.09 ± 0.13	4.37 ± 0.09	3–5	59.02	< 0.001
LESF 594	*Escovopsioides nivea*	9.10 ± 0.13	5.46 ± 0.12	3–5	48.56	< 0.001
LESF 595	*Escovopsioides nivea*	8.51 ± 0.16	4.51 ± 0.12	3–5	44.10	< 0.001
LESF 510	*Escovopsioides nivea*	8.20 ± 0.12	4.18 ± 0.07	3–5	44.03	< 0.001
LESF 591	*Escovopsioides nivea*	8.95 ± 0.16	5.87 ± 0.18	5–7	26.71	< 0.001
LESF 151	*Escovopsioides nivea*	8.19 ± 0.13	4.24 ± 0.11	5–7	16.26	< 0.001
LESF 603	*Escovopsioides nivea*	8.36 ± 0.20	4.45 ± 0.16	5–7	14.45	0.001
LESF 592	*Escovopsioides nivea*	8.36 ± 0.18	5.42 ± 0.17	5–7	14.26	0.001
LESF 599	*Escovopsioides nivea*	8.36 ± 0.18	6.00 ± 0.12	5–7	13.85	0.001
LESF 596	*Escovopsioides nivea*	8.36 ± 0.20	6.85 ± 0.19	7–10	0.84	0.370
LESF 602	*Escovopsioides* sp.	8.20 ± 0.12	6.65 ± 0.15	7–10	0.35	0.558

CGd_10_: growth area of *L. gongylophorus* in the control plates at day 10; FG_d10_: growth area of *L. gongylophorus* in the presence of *Escovopsioides* or *Escovopsis* at day 10, Int-In: inhibition interval (in days). Significant differences between CGd_10_ and FG_d10_ were verified by pairwise t-test with Bonferroni correction (*P* < 0.01)

**Fig. 2:**
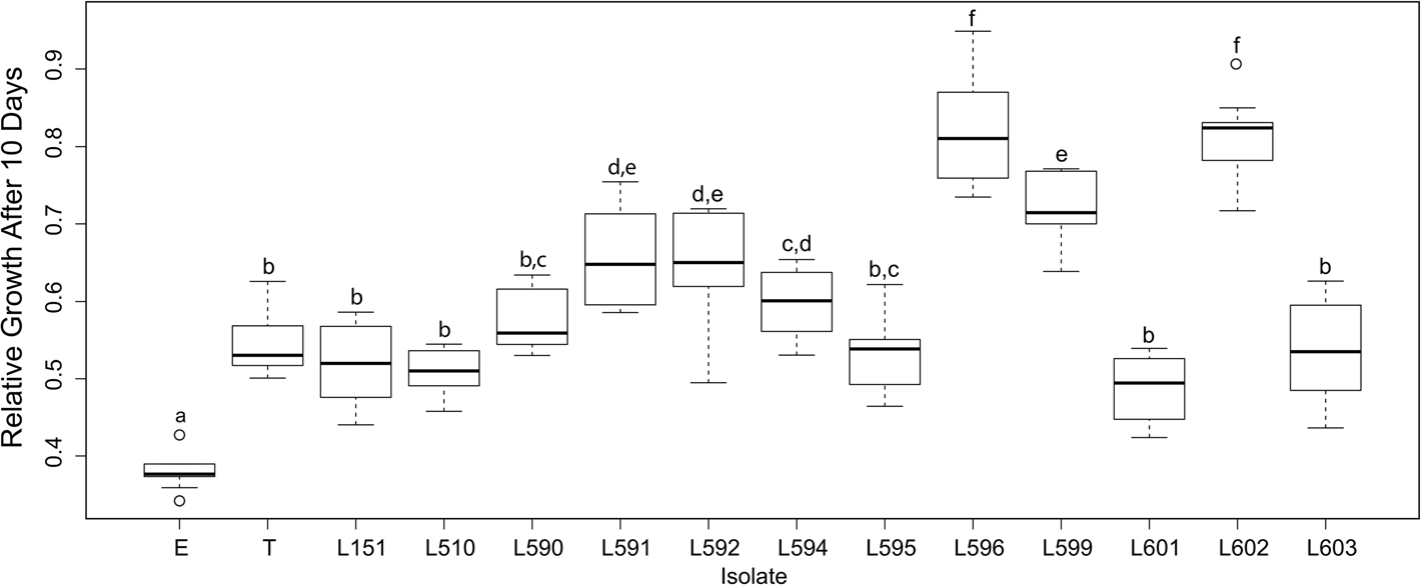
Mycelial growth of *Leucoagaricus gongylophorus*, the fungus cultivated by leaf-cutter ants, in the presence of *Escovopsisoides* isolates. The relative mycelial growth was obtained by dividing the growth area of *L. gongylophorus* towards the different *Escovopsioides* isolates in relation to the corresponding control (absence of *Escovopsioides*) after 10 days. Data followed by different letters are significantly different (*P* ≤ 0.05). E: *Escovopsis* sp. LESF 19 was used for comparison. T: type species of *Escovopsioides nivea*. L: refers to the LESF Ids of *Escovopsioides* strains listed in Table 3

After 10 days of experiment three significantly distinct groups of *Escovopsioides* isolates were observed regarding the inhibition of the mutualistic fungus (Fig. 2). Isolates LESF 596 and LESF 602, obtained from *Acromyrmex* sp. and *A. megacephala*, respectively, comprise a group of fungi that significantly inhibited the growth of the mutualistic fungus at day 10, but to a lesser extent when compared to the remaining isolates (Tukey-HSD, *P* < 0.003, Fig. 2). *Escovopsis* showed the highest inhibiton of the mutualistic fungus (Tukey-HSD, *P* < 0.001, Fig. 2).

### Bioassays on fungus garden fragments in the absence of workers

**Fig. 3:**
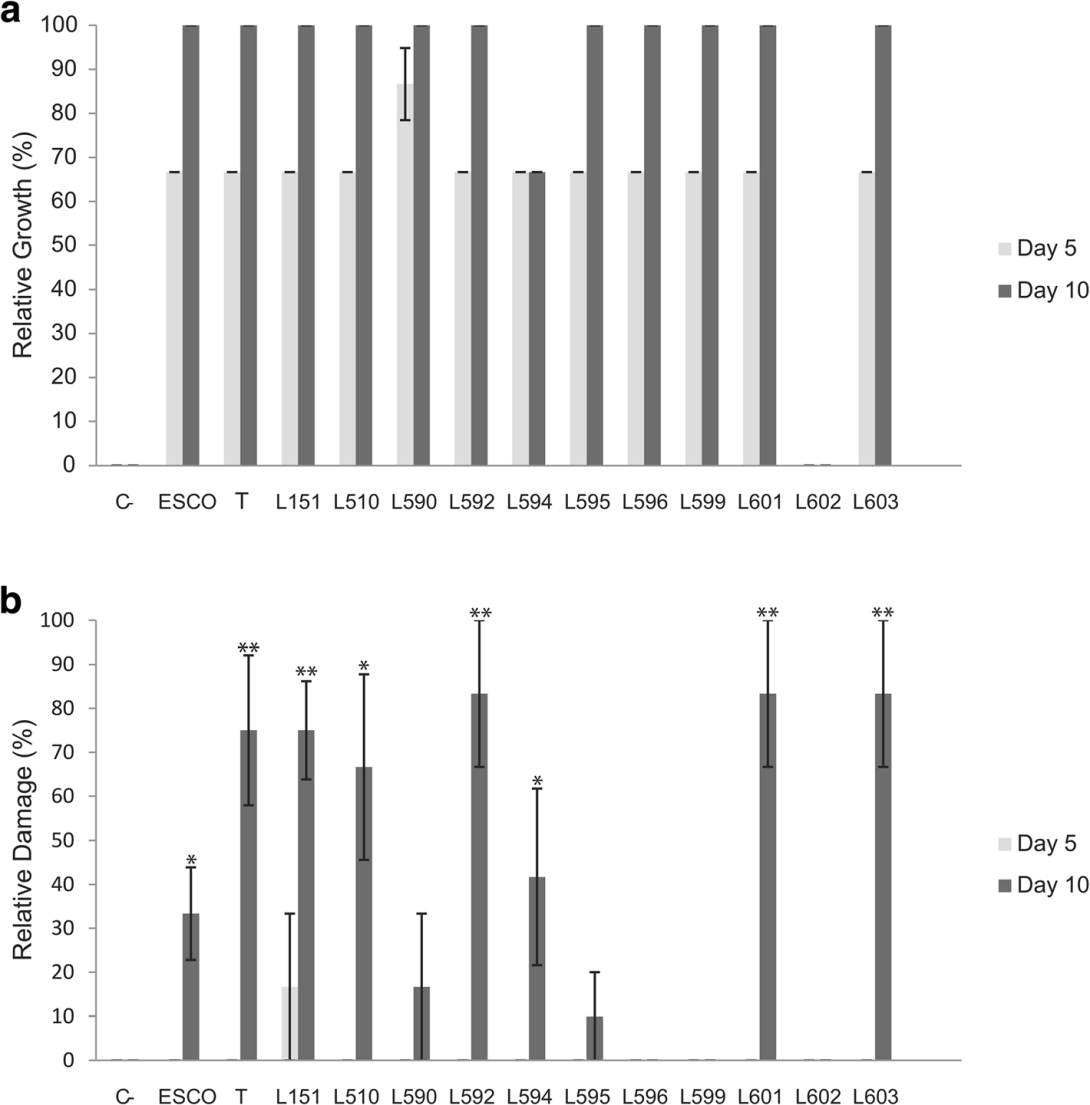
Fungus gardens of *Atta sexdens rubropilosa* treated with *Escovopsoides* conidia. (**a**) Ant-free fungus gardens and (**b**) Fungus gardens with ant workers. Light gray bars: treatments after 5 days. Dark grey bars: treatments after 10 days. The y-axis indicates relative growth of *Escovopsioides* (**a**) and damage (in %) on fungus gardens (**b**). Values are means ± standard errors of proportions of the scores in the different treatments. * and ** indicates significant damage on fungus gardens with workers *P* ≤ 0.05 and *P* ≤ 0.01, respectively. ESCO: *Escovopsis* sp. LESF 19 was used for comparison. T: type species of *Escovopsioides nivea*; C-: negative control. L followed by numbers refers to the LESF Ids listed in Table 3

Overall, *Escovopsioides* isolates and *Escovopsis* grew on fungus gardens and no differences were observed in their development (Fig. 3a and Additional file 1: Figure S1). *Escovopsioides* sp. LESF 602 was the only isolate that did not grow on the fungus gardens after 10 days (Fig. 3a). On day 10 of the experiment, 11 out of 12 *Escovopsioides* isolates presented conidiation on the fungus gardens. The exception was *E. nivea* LESF 594, which did not produce conidia on the fungus gardens.

### Effects of *Escovopsioides* on gardens with workers

We observed three main responses when inoculating garden fragments with the presence of workers. The first one was observed for the majority of *Escovopsioides* isolates along with the *Escovopsis* isolate. For this group, we recorded significant damage in the fungus gardens (*P* < 0.049, Fig. 3b). Fungus gardens treated with conidia of these fungi succumbed and most of the infected fungus gardens were removed and degraded (Additional file 1: Figure S2). We observed *Escovopsioides* growth in fragments of this substrate when inoculated on the culture medium (Table 2). However, fast-growing fungi also grew along with *Escovopsioides* and covered the Petri dishes. Because of that we could not recover *Escovopsioides* from all containers in these treatments. Thus, all data obtained from the infected fungus garden were not used in the comparative analyses with fungi isolated from the healthy fungus gardens.

**Table 2:** Presence of *Escovopsioides* in fungus gardens of *Atta sexdens rubropilosa* after 0, 5 and 10 days of treatment with conidia of this fungus. The values are means ± standard errors of garden fragments with conidia on a scale of percentage, with 0 (no conidia present on garden fragments) to 100 (conidia present on all garden fragments). Data followed by different letters are significantly different between treatments in the same day (*P* ≤ 0.05). *Escovopsis* sp. LESF 19 was used for comparison. ^T^: type species of *Escovopsioides nivea*

Isolates	Day 0	Day 5	Day 10
Control	0a	0 a	0a
LESF 596	100b	48.27 ± 14.79 b,c	44.82 ± 13.53c,d
LESF 599	100b	73.91 ± 22.75c,d	26.08 ± 9.52b,c
LESF 602	100b	33.33 ± 8.78b	4.76 ± 4.76a,b
LESF 590	100b	103.57 ± 3.57f	71.42 ± 14.28d
LESF 595	100b	84 ± 7.48d,e	60 ± 17.12d
LESF 151	100b	100f	70 ± 16.93^*^
LESF 510	100b	100f	16.66 ± 16.66^*^
LESF 592	100b	93.33 ± 6.66d,e,f	33.33 ± 15.20^*^
LESF 594	100b	82.14 ± 8.60d,e	46.42 ± 20.26^*^
LESF 601	100b	96.66 ± 3.33e,f	10 ± 10^*^
LESF 603	100b	100f	70 ± 10^*^
CBS 135749^T^	100b	100f	20 ± 10.32^*^
*Escovopsis* sp*.* LESF 19	100b	100f	100^*^

^*^Values not considered in the analysis, since they refer to *Escovopsioides* growth in waste material, not from the fungus garden

The second response consisted of observations for *E. nivea* LESF 590 and LESF 595. A moderate reduction in the frequency of recovery of these fungi was observed (Table 2). In addition, the health conditions of the fungus gardens infected with the two isolates did not differ significantly from that of the control, although a slight decrease in garden health was observed (*P* > 0.36, Fig. 3b).

Finally, the third response was observed for the isolates LESF 596 and LESF 599, where great removal of conidia was observed on day 10 (Table 2). In addition, conidia of *Escovopsioides* sp. LESF 602 was almost completely absent from the fungus gardens on day 10. Thus, fungus gardens inoculated with conidia of these three isolates were completely healthy on day 10 (*P* = 1.00, Fig. 3b).

## Discussion

Here, we showed for the first time that *E. nivea* is found in colonies of different higher attine ants from distinct geographic regions. In addition, in vitro bioassays coupled with experiments using both fungus gardens with and without tending ants showed this fungus is an effective antagonist of the mutualistic fungus cultivated by leafcutter ants.

The Bayesian inference resolved *Escovopsoides* in a monophyletic clade and as a sister group of *Escovopsis* supporting results found by Augustin et al. [18]. Most of the isolates (*n* = 20) showed low variation in the molecular markers, corroborating the identification to the type species, *E. nivea* described by Augustin et al. [18]. The only exception was *Escovopsioides* sp. LESF 602, obtained from a colony of *A. megacephala*, which diverged from the type species of the genus, suggesting a new phylogenetic species. The description of this new taxon will be provided in future studies as the quest for additional isolates of this taxon continues. Moreover, the genus *Escovopsioides* needs to be further investigated to unravel its diversity, especially in lower attine ants, from which there are no reports of its occurrence.

In the analysis we noted that the sample locality does not explain the clustering of the several *Escovopsioides* isolates. A similar result was observed by Taerum et al. [21] for *Escovopsis* isolates obtained from very distant places, such as Panama and Argentina. Moreover, these authors also did not observe a separation of *Escovopsis* that infects *Atta* colonies and others that only infect *Acromyrmex* colonies, demonstrating a high degree of parasite sharing among leafcutter ants. Similarly, we observed that *Atta*, *Acromyrmex* and *Trachymyrmex* share the same species (*E. nivea*) in their colonies.

Our data suggests that *Escovopsioides* is an antagonist of the ant mutualistic fungus. Varanda-Haifig et al. [16] raised indications that *Escovopsioides* shows inhibitory activity against the mutualistic fungus of leafcutter ants, but the authors used only two isolates obtained from ants in the genus *Acromyrmex*. The inhibitory activity of *Escovopsioides* could be verified from putative compounds produced by this fungus during hyphae-hyphae interaction [16]. Here, our experiments further support these results by assessing a more comprehensive set of isolates from diverse attine ant colonies. In addition, we also observed that *Escovopsioides* is an antagonist of the *L. gongylophorus* in its natural substrate (i.e. fungus gardens) in the absence or presence of worker ants.

In a context considering the protective role of the ants, the effect of some isolates changed significantly in comparison to the experiment in the absence of workers, because they did not cause significant damage in the fungus gardens with workers. Elizondo-Wallace and colleagues [22] observed that *Escovopsis* bearing identical *tef1* gene sequences showed marked differences in garden damage in the presence of *Atta cephalotes* workers. Moreover, Taerum et al. [21] discussed the possibility that subtle genetic differences among *Escovopsis* isolates may have a very significant impact on the virulence of the parasite in the presence of workers. This may explain why the effect of genetically similar *Escovopsioides* isolates showed marked differences in the health of fungus gardens with workers. Therefore, this observation is evidence of how essential workers are in maintaining the health of fungus gardens.

We observed an absence of significant damage in fungus gardens infected with conidia of *E. nivea* LESF 596 and LESF 599. In addition, we observed that the frequency of detection of these isolates in fungus gardens with workers after treatment was significantly reduced overtime, suggesting the infection was under control (Fig. 3). This result could be due to the protective behavior of the ants by weeding out contaminated portions of the fungus garden or removing conidia by grooming [21]. On the other hand, in treatments with isolates LESF 151, LESF 510, LESF 592, LESF 594, LESF 601, LESF 603, CBS 135749^T^ and *Escovopsis* sp. LESF 019, where severe damage was observed in the fungus garden, the partial or complete weeding of the infected fungus garden was observed (Additional file 1: Figure S2). Ants’ dumps are recognized as a decomposing material rich in microorganisms, where a distinct microbiota from that found in the fungus garden prevails [23]. Several exogenous microorganisms to the ant colony may potentially outcompete and inhibit the development of *Escovopsioides* on the culture medium [24]. This may explain why *Escovopsioides* growth was often not observed when inoculating infected garden fragments on culture medium (Table 2).

*Escovopsioides nivea* LESF 601 was isolated from a colony of the higher attine ant genus *Trachymyrmex.* This genus like *Atta* and *Acromyrmex* belongs to the higher-attines, but cultivates a different fungus from that of the leafcutter ants (data not shown). The effect of LESF 601 in the dual-culture experiments against *L. gongyloporus* as well as on the fungus garden in the absence and presence of workers was indistinguishable from the most virulent isolates obtained from leafcutter ants (Fig. 3). Taerum et al. [21] observed the growth of *Escovopsis* isolates associated with *Trachymyrmex* ants on fungus gardens of leafcutter ants. Therefore, our results may suggest that *Escovopsioides* appears not to present specificity to its host, when considering leafcutter ants and non-leafcutter ants. However, this hypothesis should be tested further since in our experiments we challenged *Escovopsioides* only with one host fungus (*L. gongylophorus*). Thus, specificity and the variations observed regarding the antagonism of different *Escovopsioides* isolates may differ when using other fungal hosts such as those from non-leafcutter ants.

*Escovopsioides* sp. LESF 602 is the only isolate obtained from a lower attine ant colony, *A. megacephala*. This ant is distinguished from other species of *Apterostigma* by cultivating *L. gongylophorus*, the same mutualistic fungus cultivated by leafcutter ants [25]. Because this ant species cultivates this fungus, it was expected that the *Escovopsioides* found in this colony would cluster with all other *Escovopsioides* isolates found in leafcutter ant colonies. Contrary to the expected, LESF 602 was the most distinguished isolate in our phylogeny. Additionally, this isolate was the only one that did not demonstrate any effects in the fungus gardens both in the absence and in the presence of workers. Caldera and Gerardo [26] and Meirelles et al. [27] suggested that *Escovopsis* isolates are more adapted to particular geographic regions (local specialization). In the case of *A. megacephala*, this is a rare species found only in the Amazon region [25] and although these authors described the mutualistic fungus *L. gongylophorus* as the *A. megacephala* cultivar, this fungus belongs to a unique clade. Therefore, Gerardo et al. [28] appointed that different *Escovopsis* isolates attack only specific hosts through chemotaxis with the host also being able to protect itself with antibiotics against no specific *Escovopsis*. This may indicate certain specificity in *Escovopsioides* for the mutualistic fungus of this ant. In this sense, this could explain why LESF 602 is different than the other isolates, and the absence of damages in the mutualistic fungus used in the bioassays. However, this suspisions can be only confirmed when more isolates of this species become available.

## Conclusions

Here, we showed low genetic diversity among *Escovopsioides* isolates from higher attine ants, showing a wide distribution of *E. nivea* in different ant colonies in distinct localities. More sampling efforts for *Escovopsioides* isolates may expand the diversity of this genus especially those associated with lower attine ants. The present study also indicated that *Escovopsioides* is an antagonist of the fungus gardens of leafcutter ants.

## Methods

### Fungi examined

*Escovopsioides* isolates were obtained from fungus gardens of different leafcutter and non-leafcutter ant species collected in previous studies [18–20, 29, 30]. This collection comprises 20 isolates obtained from *Atta sexdens*, *Atta capiguara*, *Atta cephalotes*, *Acromyrmex balzani*, *Acromyrmex heyeri*, *Acromyrmex lundi* and *Trachymyrmex tucumanus*, in addition to the type species of *E. nivea* obtained from *Acromyrmex subterraneus subterraneus* (Table 3). Also, one additional isolate was obtained from a colony of the non-leafcutter ant *Apterostigma megacephala* found in Parauapebas, Pará, Brazil (Table 3). All isolates displayed the described morphological characteristics of the genus *Escovopsioides*: hyaline colonies on PDA, terminal and intercalary vesicles bearing lageniform phialides, hyaline conidia in long chains, aleuroconidia and chlamydospore-like structures [18].

**Table 3:** *Escovopsioides* isolates (*n* = 21) obtained from fungus gardens of attine ants and used in the present study

Isolate ID^a^	Ant species	City/State	Coordinates of ant colonies	Colony Id^b^	Reference
LESF 57	*Atta sexdens rubropilosa*	Corumbataí/SP	22°17′22″S 47°39′23″W	Colony 4	[29]
LESF 151^c^	*Atta sexdens rubropilosa*	Corumbataí/SP	22°17′22″S 47°39′23″W	Colony 9	[29]
LESF 159	*Atta sexdens rubropilosa*	Corumbataí/SP	22°17′22″S 47°39′23″W	Colony 7	[29]
LESF 510^c^	*Atta sexdens rubropilosa*	Botucatu/SP	22°54′28.44″S 48°18′55.68″W	JSP130130–04	[20]
LESF 587	*Atta cephalotes*	Camacan/BA	15°23′18.18″S 39°33′30.54″W	BMSR120704–01	[19]
LESF 588	*Atta cephalotes*	Camacan/BA	15°25′32.28″S 39°32′48.06″W	BMSR120804–01	[19]
LESF 589	*Atta cephalotes*	Camacan/BA	15°23′14.82″S 39°33′28.38″W	BMSR120702–01	[19]
LESF 590^c^	*Atta cephalotes*	Camacan/BA	15°23′15.18″S 39°33′28.02″W	BMSR130220–01	[19]
LESF 591^c^	*Atta capiguara*	Botucatu/SP	22°54′26.64″S 48°18′29.22″W	JSP130307–03	[20]
LESF 592^c^	*Acromyrmex balzani*	Camacan/BA	15°22′50.34″S 39°34′3.54″W	ARFVG110517–01	–
LESF 593	*Atta cephalotes*	Camacan/BA	15°23′17.76″S 39°33′22.26″W	BMSR120703–01	[19]
LESF 594^c^	*Atta cephalotes*	Camacan/BA	15°25′32.28″S 39°32′48.06″W	BMSR120804–01	[19]
LESF 595^c^	*Atta cephalotes*	Camacan/BA	15°23′14.82″S 39°33′28.38″W	BMSR120702–01	[19]
LESF 596^c^	*Acromyrmex lundi*	Chuvisca/RS	30°50′10.2″S 51°55′10.4″W	AOMB110904–02	[30]
LESF 597	*Atta cephalotes*	Camacan/BA	15°23′29.7″S 39°33′31.32″W	BMSR120703–02	[19]
LESF 598	*Atta cephalotes*	Camacan/BA	15°23′17.76″S 39°33′22.26″W	BMSR120703–01	[19]
LESF 599^c^	*Acromyrmex heyeri*	Sentinela do Sul/RS	30°37.57′9.0″S 51°33′18.2″W	AOMB100904–03	[30]
LESF 601^c^	*Trachymyrmex tucumanus*	Rio Claro/SP	22°23′45.84″S 47°32′41.58″W	SES080402–04	–
LESF 602^c^	*Apterostigma megacephala*	Parauapebas/PA	6°3′46.98″S 50°3′27.54″W	JSC110910–03	–
LESF 603^c^	*Atta sexdens rubropilosa*	Corumbataí/SP	22°17′22″S 47°39′23″W	Colony 12	[29]
CBS 135749^Tc^	*Acromyrmex subterraneus subterraneus*	Viçosa/MG	20°44′31.71″S 42°52′43.83″W	–	[18]

^a^LESF: Fungal collection of the Laboratory of Ecology and Fungal Systematics (UNESP, Rio Claro, Brazil)

^b^Isolates LESF 589 and LESF595 were obtained from the same ant colony but in different collecting years, 2013 and 2012, respectively

^c^Isolates used in the dual-culture assays

^T^Type species

The isolates are kept as conidia suspensions at − 80 °C (in glycerol 10%) and in distilled water at 10 °C in the collection of the Laboratory of Fungal Ecology and Systematics (LESF), Rio Claro, São Paulo State, Brazil. All isolates were revived from stocks by inoculating preserved conidia on PDA medium and incubated for 7 days at 25 °C, in darkness. All isolates examined were obtained as monosporic cultures.

### Molecular characterization of *Escovopsioides*

Genomic DNA was extracted by the CTAB method from cultures grown on malt agar 2% (MA2%) for 5 days, in darkenss [31]. The genes encoding for *tef1* (primers: EF6-20F and EF6A-1000R), LSU (primers: CLA-F and CLA-R) as well as ITS (primers: ITS5 and ITS4) were amplified for all isolates. For *tef1*, 1 μL of diluted genomic DNA (1:100) was used as template for amplification using illustra™ PureTaq Ready-To-Go PCR Beads (GE Healthcare) under the following conditions: an initial denaturation step at 94 °C for 2 min, followed by 15 cycles at 94 °C for 30 s and initial annealing temperature at 65 °C decreasing by 1 °C per touchdown cycle and final extension at 72 °C for 1 min. A second PCR step was performed: 94 °C for 30 s, followed by 35 cycles at 94 °C for 30 s, 48 °C for 30 s and a final extension step at 72 °C for 1 min. For ITS, 2 μL of the diluted genomic DNA (1:100) were used as template for PCR, following the conditions described in Schoch et al. [32]: 94 °C for 3 min, followed by 35 cycles at 94 °C per 1 min, 55 °C for 1 min and a final extension step at 72 °C for 2 min. For LSU, 2 μL of diluted genomic DNA (1:100) were used for PCR, following the conditions described in Augustin et al. [18]: 95 °C for 2 min, 40 cycles of 30 s at 95 °C, 60 s at 62 °C, 90 s at 72 °C and 5 min of extension at 72 °C. PCR products were stained with GelRed™ (Biotium) and visualized under UV after electrophoresis in 1% agarose gel.

Amplicons were purified with the Wizard® SV Gel and PCR Clean-up System Kit (Promega) following the manufacturer’s protocol. Samples were analyzed in NanoDrop™ 2000 (Thermo Fisher Scientific) and 20 ng of DNA were sequenced using BigDye™ Terminator v. 3.1 Cycle Sequencing Kit (Thermo Fisher Scientific) following the manufacturer’s instructions. Due to high GC content in the ITS region of *Escovopsioides*, 5% of DMSO was added to the sequencing reactions. Forward and reverse sequences were generated in ABI 3500 (Life Technologies) and assembled into contigs in BioEdit v.7.1.3 [33]. Sequences generated in the present study were deposited at NCBI-GenBank (Additional file 1: Table S1).

### Phylogenetic analysis

In addition to the sequences generated in this study, the sequences of the type species (*E. nivea*) along with of six described *Escovopsis* species and eight fungi belonging to the Hypocreales (*Hypomyces*, *Trichoderma* and *Lecanicillium*) were retrived from the NCBI-GenBank. Thus, the whole dataset used in this study comprised sequences of 37 fungi (Additional file 1: Table S1).

For the phylogenetic analysis, sequences were aligned in MAFFT [34]. Sequences of *tef1*, ITS and LSU were concatenated in Winclada v.1.00.08. Bayesian inference was used as the reconstruction algorithm. The nucleotide substitution model GTR + G and GTR + I + G was selected for *tef1* and ITS/LSU, respectively, using AIC in jModeltest2 [35] with a confidence interval of 95%. Thus, partitioned independent analyses of the dataset were performed in MrBayes [36] each with three heated chains and one cold chain. MCMC sampling in each analysis occurred up to one million generations. Convergence of heated and cold chains occurred when the standard deviation of split frequencies was below 0.01; then the first 25% of generations were discarded as burn-in.

### Dual-culture experiments

We performed in vitro tests to verify the antagonistic potential of *Escovopsioides* towards the fungus cultivated by leafcutter ants. We selected 13 out of 21 *Escovopsioides* isolates (Table 3), based on the following criteria: i) any polymorphism found in the *tef1* gene; ii) different associated ant species; iii) geographical location (i.e. different cities and collection sites). Two of the selected isolates (LESF 596 and LESF 599) were already evaluated for their interactions with the fungal cultivar in the study of Varanda-Haifig et al. [16]. However, we replicated this assay to compare with additional *Escovopsioides* isolates.

The fungus *Leucoagaricus gongylophorus* FF2006 cultivated by the ant species *Atta sexdens rubropilosa* was used in the dual-cultural experiments. This strain is maintained on agar slants by successive transfers every 24 days at 25 °C. Production of gongylidia by this strain is checked in every transfer cycle. This fungus was grown on PDA medium and incubated at 25 °C for 14 days, in darkeness. After this period, mycelial fragments (8 mm in diameter) were transfered to new Petri plates (90 × 15 mm) also containing PDA at a distance of 1.5 cm from the border. These plates were incubated at 25 °C for 14 days, in darkeness. Then, mycelium fragments (8 mm in diameter) of *Escovopsioides*, previously incubated on PDA, were inoculated at a distance of 3 cm apart from the mutualistic fungus. To compare the effects of *Escovopsioides* on the mycelium growth of the fungal cultivar with the effects caused by other fungi, we used one *Escovopsis* sp. isolate (LESF 19) from the fungus gardens of *Atta sexdens rubropilosa* (Botucatu, Brazil). This isolate was obtained by inoculating small pieces of the fungus gardens on PDA supplemented with chloramphenicol. Control plates were inoculated with the same mutualistic fungus, however, no *Escovopsioides* or *Escovopsis* were inoculated. All plates were incubated for 14 days at 25 °C, in darkness. Each combination of *Escovopsioides, Escovopsis* and the mutualistic fungus were carried out in ten plates. Experimental and control plates were scanned at intervals of 0, 2, 3, 5, 7, 10 days on a HP Deskjet F2050 scanner. Images were used to measure the area of colony growth (cm^2^) of the mutualistic fungus in ImageJ v. 1.38 [37].

The mycelial growth areas of *L. gongylophorus* in the dual-culture experiments were analyzed using two-way mixed ANOVA, with fungal isolates (*Escovopsioides* and *Escovopsis*) versus the control as the between-subjects variable, and time (days) as the within-subjects variable, thus considering the repetead measurements of time in the treatments. The data were checked for normality and homogeneity of variances, using the Shapiro-Wilk and Levene tests, respectively. Due to the violations of these criteria, data were transformed using the log or square root when necessary. Multiple comparisions with different filamentous fungi were performed using t-test with Bonferroni correction.

For comparison between isolates, the mycelial growth area of *L. gongylophorus* in contact with *Escovopsioides* isolates was divided by the mean area of its control at day 10. The data were checked for normality and homogeneity of variances. One-way ANOVA was performed with the area obtained at day 10 between all tested fungi, with comparisons between the different treatments performed using post-hoc Tukey-HSD test. Statistical analyses were performed in R v. 2.12.1 [38].

### Bioassays on fungus garden fragments in the absence of workers

*Leucoagaricus gongylophorus* is cultivated in attine ant colonies in fungus gardens. To determine whether *Escovopsioides* can develop on the fungus garden without the possible protective effects of the workers, we performed bioassays using fragments of this substrate free of ants. Fungus garden fragments were obtained from one mature and healthy *A. sexdens rubropilosa* colony maintained at the Center for the Studies of Social Insects (UNESP, Rio Claro). Fungus garden fragments of 2 cm^3^ deprived of ants and brood were placed in sterile Petri dishes with moistened cotton at the edges (following Elizondo-Wallace et al. [22]). Before carring out the experiments, plates were kept for 1 day at 25 °C to search for ants that possibly remained in the fragments.

We prepared a 10 mL suspension of 0.05% Tween 80 with mycelial mass and conidia of each of the 12 *Escovopsioides* (isolate LESF 591 was not used in this experiment) and one *Escovopsis* isolate used in the dual-culture experiments. This suspension was filtered two to three times following the method by Newmeyer [39] to separate the conidia from the hyphal fragments present in the suspension. Then, the suspension was diluted to 10^5^ to 2.10^5^ conidia mL^−1^determined in a Neubauer chamber. Aliquots of 100 μL of conidia suspensions were inoculated on the surface of the garden fragment using a micropipette. The same amount of sterile 0.05% Tween 80 solution was added on the garden surface in the negative control. Petri dishes with fungus garden were kept at 25 °C for up to 10 days, with five dishes used for each treatment and five dishes for each respective control. The possible development of hyphae of the inoculated fungi was observed daily on a stereomicroscope (EZ4, Leica). *Escovopsioides* conidiation on the fungus gardens was observed by taking mycelium fragments growing on the gardens and mounted in water and observed under the microscope (DM500, Leica).

Growth and conidiation data on the fungus gardens were scored as: no growth (score 0), growth observed only on the stereomicroscope (score 1), macroscopic growth (score 2) and conidiation (score 3) over 10 days of monitoring (Additional file 1: Figure S1). We observed conidiation only after fungal macrocospic growth on the fungus gardens. The total sum of the scores of five Petri dishes on each day was obtained and transformed in terms of percentage of the maximum possible total for direct comparisons.

### Effects of *Escovopsioides* on gardens with workers

Ant workers have an important role in colony defense against pathogens and unwanted microbes in the fungus gardens [40]. Thus, we performed experiments to check the influence of workers in fungus gardens inoculated with conidia of the same fungi used in the bioassays on fungus garden in the absence of ant workers (12 *Escovopsioides* isolates and 1 *Escovopsis* isolate).

Bioassays using queen-right ant colonies are challenging due to the large number of colonies this type of experiment would demand. Thus, we performed bioassays with gardens fragments containing workers to assess the effects of the fungal isolates in vivo. Although this experimental set up does not represent what occurs in natural colonies, it showed indicative information about the defensive action of workers against the tested fungi. This bioassay was adapted following the method by Elizondo-Wallace et al. [22]. Plastic containers with a tiny layer of plaster in the bottom were used to avoid desiccation of the fungus gardens. About 20 cm^3^ of fungus garden from the same colony used in the previous experiment were placed in the plastic containers. The experimental set up comprised a total of 84 containers, so that treatments with conidia of the 13 fungi and the control had six containers each. We selected worker ants with cephalic capsule diameter between 1.0 and 1.6 mm [41], then 50 of these workers were placed in each container. Workers with less than 1.0 mm were not counted, and workers with more than 1.6 mm were excluded. This procedure was adopted to allow homogeneity between the six containers from each treatment as well as between the treatments, because workers in the interval between 1.0 and 1.6 mm have a significative cleaning activity [41]. The inner surface of the container was coated with Teflon® to prevent the ants from escaping.

The containers were incubated at 25 °C for 3 days to stabilize the fungus gardens. Conidia suspensions were obtained as described above. A total of 1 mL of conidia suspension was sprayed on the surface of the fungus gardens using a sprayer. Fungus gardens in the negative control were sprayed with only1 mL of sterile 0.05% Tween 80 solution.

The experiments were monitored after 0, 5 and 10 days of inoculation to check the health of the fungus gardens. This parameter was based on a scoring system, in which healthy gardens received score 0, partially degraded gardens received score 1 and completely degraded (“infected”) gardens received score 2. The ant removal of fungus gardens pieces and allocation to the refuse dumps was the main sign of garden deterioration we examined (see Additional file 1: Figure S2). The scores obtained from the six containers of each experiment, on the 3 days of observation, were compared using Friedman test.

To verify the presence of the *Escovopsioides* isolates on the fungus gardens on days 0, 5 and 10 after treatment, fragments were removed from gardens and inoculated on MA2% supplemented with 150 μg mL^− 1^ of chloramphenicol. Plates were incubated at 25 °C for 7 days, in darkness. Five plates with one garden fragment were used for each one of the six containers from each treatment, totaling 30 plates per treatment. Fragments with positive growth were counted and the long-term presence of the inoculated fungi in the fungus gardens was tested using Fisher’s Exact test. In this analysis we compared the proportion of frangments infected with fungal conidia (*Escovopsioides* and *Escovopsis*) versus the control without any conidia in each day of experiment. We also compared the different treatments with inoculated fungi between each other in each day of experiment.

## Additional file

Additional file 1: **Table S1.** Sequences used in the phylogenetic analyses and their associated metadata. **Figure S1.** Fungus gardens of *Atta sexdens rubropilosa* after 10 days of treatment with fungal spores. Fungus gardens were sampled from a mature colony and left in Petri dishes without any ants. (A) Garden not sprayed with spores (control); (B) Garden overgrown by *Escovopsioides nivea* LESF 603; (C) Healthy garden after treatment with spores of *Escovopsioides* sp. LESF 602 and (D) Garden overgrown by *Escovopsis* sp. LESF 19. **Figure S2.** Criteria used to designate health conditions of fungus garden in the bioassays using fungus gardens of *Atta sexdens rubropilosa*. (A) healthy garden; (B) garden in process of deterioration and (C) dead garden after 10 days of experiment. (DOCX 1760 kb)

## Abbreviations

AIC: Akaike Information Criterion
CTAB: cetyltrimethyl ammonium bromide
ITS: internal transcribed spacer region
LSU: gene that encodes for the large subunit of the ribosomal RNA
MCMC: Markov Chain Monte Carlo
PCR: polymerase chain reaction
PDA: potato-dextrose agar
*tef1*: gene that encodes for the elongation factor 1-alpha
