# Supplementary material for: Escovopsioides as a fungal antagonist of the fungus cultivated by leafcutter ants
Source: BMC Microbiol. 2018 Oct 10;18:130. doi: 10.1186/s12866-018-1265-x (PMC6180628; doi:10.1186/s12866-018-1265-x)

**Additional file 1**

***Escovopsioides* as a fungal antagonist of the fungus cultivated by leafcutter ants**

Julio Flavio Osti^1^, Andre Rodrigues^1,2*^

^1^ Department of Biochemistry and Microbiology, São Paulo State University (UNESP), Rio Claro, Brazil.

^2^ Center for the Studies of Social Insects, São Paulo State University (UNESP), Rio Claro, Brazil.

The supplementary material includes Table S1 and Figures S1-S2.

* Corresponding author

Andre Rodrigues ([andrer@rc.unesp.br](mailto:andrer@rc.unesp.br))

Universidade Estadual Paulista, UNESP, Câmpus de Rio Claro

Avenida 24-A, 1515, Rio Claro, SP, 13506-900, Brazil

Phone #: +55 19 3526-4364

**Table S1.** Sequences used in the phylogenetic analyses and their associated metadata.

| **Species** | **Strain ID** | **Origin** | **Habitat** | **GenBank accession numbers** | | | **References** |
| --- | --- | --- | --- | --- | --- | --- | --- |
|  |  |  |  | **ITS** | ***tef*1** | ***LSU*** |  |
| *Escovopsis weberi* | CBS 810.71 | Brazil | Fungus gardens of leaf-cutting ant | KF293285 | KF240730 | KF29328 | [1, 2] |
| *Escovopsis*  *microspora* | CBS 135751^T^ | Brazil | Fungus gardens of *Acromyrmex subterraneus molestans* | JQ815076 | KJ935030 | KF293284 | [1, 3] |
| *Escovopsis moelleri* | CBS 135748^T^ | Brazil | Fungus gardens of *Acromyrmex subterraneus molestans* | JQ815077 | JQ855712 | JQ855715 | [1] |
| *Escovopsis lentecrescens* | CBS 135750^T^ | Brazil | Fungus gardens of *Acromyrmex subterraneus subterraneus* | JQ815079 | JQ855714 | JQ855717 | [1] |
| *Escovopsis aspergilloides* | CBS 423.93^T^ | Brazil | Fungus gardens of *Trachymyrmex ruthae* | KF293287 | AY172632 | KF293283 | [1, 4] |
| *Escovopsis kreiselii* | CBS 139320^ET^ | Brazil | Fungus gardens of  *Mycetophylax morschi* | KJ808767 | KJ808766 | KJ808765 | [3] |
| *Escovopsis trichodermoides* | CBS 137343^T^ | Brazil | Fungus gardens of *Mycocepurus goeldii* | KJ485699 | KF033128 | MF116052 | [5], This study |
| *Cladobotryum protrusum* | TFC 201316 | Madagascar | agaric | FN859413 | FN868732 | FN859414 | [6] |
| *Cladobotryum asterophorum* | CBS 676.77^T^ | Japan | agaric | NR_111426 | FN868712 | AJ583469 | [6] |
| *Cladobotryum semicircular* | CBS 705.88^T^ | Cuba | agaric | FN859417 | FN868735 | FN859417 | [6] |
| *Hypomyces samuelsii* | TFC 2007-23 | Peru | agaric | FN859451 | FN868769 | FN859451 | [6] |
| *Hypomyces sanuelsii* | C.L.L. 7259 | West Indies | Agaric | FN859445 | FN868764 | FN859445 | [6] |
| *Trichoderma avellaneum* | CTR 77-155 | USA | - | DQ020000 | AY225857 | EU710767 | [7-9] |
| *Trichoderma atroviride* | P1 | - | soil, mycoparasite | Z48812 | EF581849 | EF591763 | [10, 11] |
| *Lecanicillium antillanum* | CBS 350.85^ET^ | Cuba | Agaric | NR_111097 | DQ522350 | AF339536 | [12-14] |
| *Escovopsioides nivea* | CBS 135749^T^ | Viçosa-MG-Brazil | Fungus gardens of *Acromyrmex subterraneus subterraneus* | JQ815078 | JQ855713 | JQ855716 | [1] |
| *Escovopsioides nivea* | LESF 57 | Corumbataí-SP-Brazil | Fungus gardens of *Atta sexdens rubropilosa* | MF116012 | MF140947 | MF116032 | This study |
| *Escovopsioides nivea* | LESF 151 | Corumbataí-SP-Brazil | Fungus gardens of *Atta sexdens rubropilosa* | MF116013 | MF140948 | MF116033 | This study |
| *Escovopsioides nivea* | LESF 159 | Corumbataí-SP-Brazil | Fungus gardens of *Atta sexdens rubropilosa* | MF116014 | MF140949 | MF116034 | This study |
| *Escovopsioides nivea* | LESF 510 | Botucatu-SP-Brazil | Fungus gardens of *Atta sexdens rubropilosa* | MF116015 | MF140950 | MF116035 | This study |
| *Escovopsioides nivea* | LESF 587 | Camacan-BA-Brazil | Fungus gardens of *Atta cephalotes* | MF116016 | MF140951 | MF116036 | This study |
| *Escovopsioides nivea* | LESF 588 | Camacan-BA-Brazil | Fungus gardens of *Atta cephalotes* | MF116017 | MF140952 | MF116037 | This study |
| *Escovopsioides nivea* | LESF 589 | Camacan-BA-Brazil | Fungus gardens of *Atta cephalotes* | MF116018 | MF140953 | MF116038 | This study |
| *Escovopsioides nivea* | LESF 590 | Camacan-BA-Brazil | Fungus gardens of *Atta cephalotes* | MF116019 | MF140954 | MF116039 | This study |
| *Escovopsioides nivea* | LESF 591 | Botucatu-SP-Brazil | Fungus gardens of *Atta capiguara* | MF116020 | MF140955 | MF116040 | This study |
| *Escovopsioides nivea* | LESF 592 | Camacan-BA-Brazil | Fungus gardens of *Acromyrmex* sp. | MF116021 | MF140956 | MF116041 | This study |
| *Escovopsioides nivea* | LESF 593 | Camacan-BA-Brazil | Fungus gardens of *Atta cephalotes* | MF116022 | MF140957 | MF116042 | This study |
| *Escovopsioides nivea* | LESF 594 | Camacan-BA-Brazil | Fungus gardens of *Atta cephalotes* | MF116023 | MF140958 | MF116043 | This study |
| *Escovopsioides nivea* | LESF 595 | Camacan-BA-Brazil | Fungus gardens of *Atta cephalotes* | MF116024 | MF140959 | MF116044 | This study |
| *Escovopsioides nivea* | LESF 596 | Camacan-BA-Brazil | Fungus gardens of *Acromyrmex* sp. | MF116025 | MF140960 | MF116045 | This study |
| *Escovopsioides nivea* | LESF 597 | Camacan-BA-Brazil | Fungus gardens of *Atta cephalotes* | MF116026 | MF140961 | MF116046 | This study |
| *Escovopsioides nivea* | LESF 598 | Camacan-BA-Brazil | Fungus gardens of *Atta cephalotes* | MF116027 | MF140962 | MF116047 | This study |
| *Escovopsioides nivea* | LESF 599 | Sentilena do Sul-RS-Brazil | Fungus gardens of *Acromyrmex heyeri* | MF116028 | MF140963 | MF116048 | This study |
| *Escovopsioides nivea* | LESF 601 | Rio Claro-SP-Brazil | Fungus gardens of *Trachymyrmex* sp. | MF116029 | MF140964 | MF116049 | This study |
| *Escovopsioides* sp. | LESF 602 | Parauapebas-PA-Brazil | Fungus gardens of *Apterostigma megacephala* | MF116030 | MF140965 | MF116050 | This study |
| *Escovopsioides nivea* | LESF 603 | Rio Claro-SP-Brazil | Fungus gardens of *Atta sexdens* | MF116031 | MF140966 | MF116051 | This study |

^ET^: ex-type species

^T^: type species

**References**

1. Augustin JO, Groenwald JZ, Nascimento RJ, Mizburti ESG, Barreto RW, Elliot SL, Evans HC (2013) Yet more "weeds" in the gardens: fungal novelties from nests of leaf-cutting ants. PLoS One 8:e82265

2. Meirelles LA, Mendes TD, Solomon SE, Bueno OC, Pagnocca FC,bRodrigues A (2014) Broad *Escovopsis* inhibition activity of *Pseudonocardia* associated with *Trachymyrmex* ants. Environ Microbiol Rep 6:389–395.

3. Meirelles, LA, Montoya QV, Solomon SE, Rodrigues A (2015) New light on the systematics of fungi associated with attine ant gardens and the description of *Escovopsis kreiselii* sp. nov. PLoS One 10:e0112067

4. Currie CR, Wong B, Stuart AE, Schultz TR, Rehner SA, Mueller UG, Sung GH, Spatafora JW, Straus, NA (2003) Ancient tripartite coevolution in the attine ant–microbe symbiosis. Science 299:386–285

5. Masiulionis VE, Cabelo MN, Seifert KA, Rodrigues A, Pagnocca FC (2015) *Escovopsis trichodermoides* sp. nov. isolated from a nest of the lover attine ant *Mycocepurus goeldii*. Anton Leeuw 107:731-40

6. Poldmaa,K. Tropical species of *Cladobotryum* and *Hypomyces* producing red pigments (2011) Stud Mycol 68:1-34

7. Samuels GJ (2005) Changes in taxonomy, occurrence of the sexual stage and ecology of *Trichoderma* sp. Phytopathology 96:195-206

8. Chaverri P, Castlebury LA, Overton BE, Samuels GJ (2003) *Hypocrea*/*Trichoderma*: species with conidiophore elongations and green conidia *Mycologia* 95:1100–1140

9. Jaklitsch WM, Poldmaa K, Samuels GJ (2008) Reconsideration of Protocrea (Hypocreales, Hypocreaceae). Mycologia 100:962-984

10. Schlick A, Kuhls K, Meyer W, Lieckfeldt E, Borner T, Messner K (1994) Fingerprinting reveals gamma-ray induced mutations in fungal DNA: implications for identification of patent strains of *Trichoderma harzianum*. Curr Genet 26:74-78

11. Brunner K, Omann M, Pucher ME, Delic M, Lehner SM, Domnanich P, Kratochwill K, Druzhinina I, Denk D, Zeilinger S (2008) *Trichoderma* G protein-coupled receptors: functional characterisation of a cAMP receptor-like protein from *Trichoderma atroviride*. Curr Genet 54: 283-299

12. Zare R, Gams W, Culham A (2000) A revision of *Verticillium* sect. *Prostrata*. I. Phylogenetic studies using ITS sequences. Nova Hedwigia 71: 465-480

13. Spatafora JW, Sung GH, Sung JM, Hywel-Jones NL, White JFJr (2007) Phylogenetic evidence for an animal pathogen origin of ergot and the grass endophytes. Mol Ecol 16:1701-1711

14. Sung,GH, Spatafora JW, Zare R, Hodge KT, Gams W (2001) A revision of *Verticillium* sect. *Prostrata*. II. Phylogenetic analyses of SSU and LSU nuclear rDNA sequences from anamorphs and teleomorphs of the Clavicipitaceae. Nova Hedwigia 72: 311-328.

**Figure S1** Fungus gardens of *Atta sexdens rubropilosa* after 10 days of treatment with fungal spores. Fungus gardens were sampled from a mature colony and left in Petri dishes without any ants. (A) Garden not sprayed with spores (control); (B) Garden overgrown by *Escovopsioides nivea* LESF 603; (C) Healthy garden after treatment with spores of *Escovopsioides* sp. LESF 602 and (D) Garden overgrown by *Escovopsis* sp. LESF 19.

**
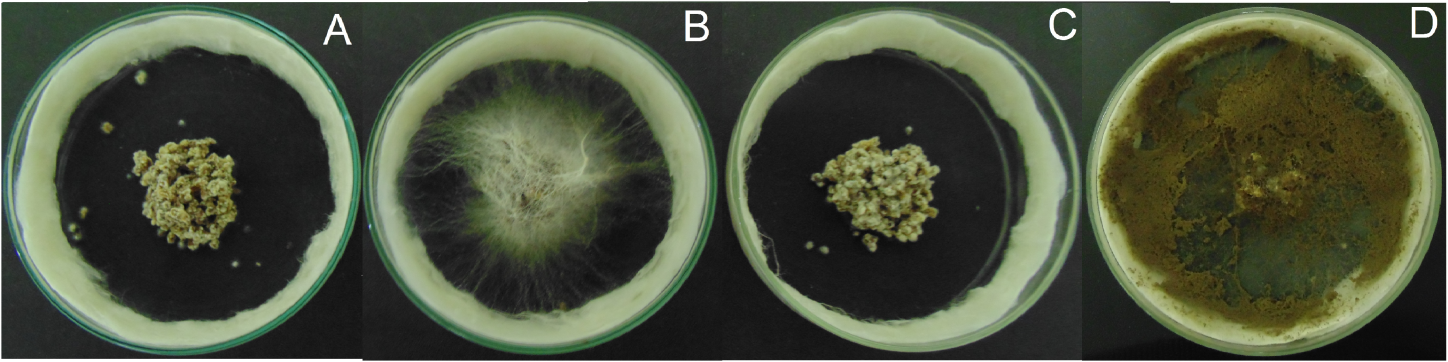
**

**Figure S2** Criteria used to designate health conditions of fungus garden in the bioassays using fungus gardens of *Atta sexdens rubropilosa*. (A) healthy garden; (B) garden in process of deterioration and (C) dead garden after 10 days of experiment.


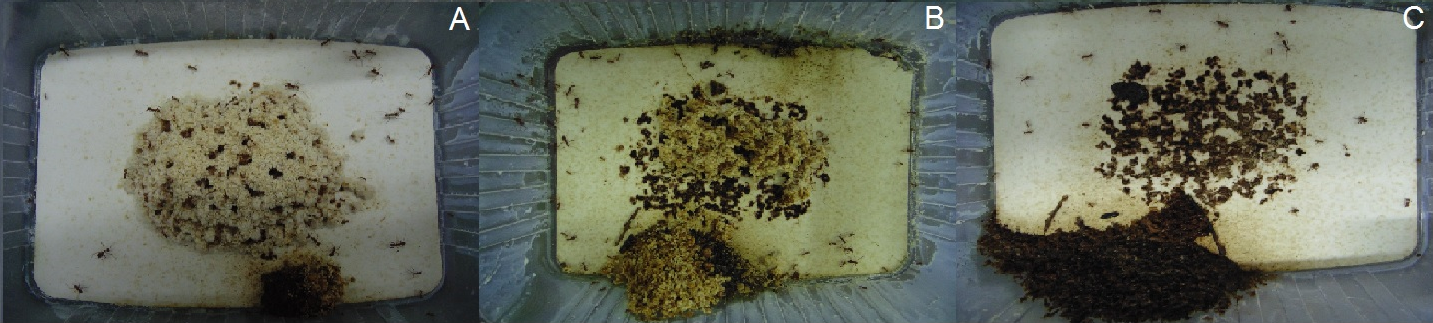

Supplement: Supplementary file 1 — Table S1. Sequences used in the phylogenetic analyses and their associated metadata. Figure S1. Fungus gardens of Atta sexdens rubropilosa after 10 days of treatment with fungal spores. Fungus gardens were sampled from a mature colony and left in Petri dishes without any ants. (A) Garden not sprayed with spores (control); (B) Garden overgrown by Escovopsioides nivea LESF 603; (C) Healthy garden after treatment with spores of Escovopsioides sp. LESF 602 and (D) Garden overgrown by Escovopsis sp. LESF 19. Figure S2. Criteria used to designate health conditions of fungus garden in the bioassays using fungus gardens of Atta sexdens rubropilosa. (A) healthy garden; (B) garden in process of deterioration and (C) dead garden after 10 days of experiment. (DOCX 1760 kb) [file 12866_2018_1265_MOESM1_ESM.docx]
